# Novel Insights into the Dermal Bioaccessibility and Human Exposure to Brominated Flame Retardant Additives in Microplastics

**DOI:** 10.1021/acs.est.3c01894

**Published:** 2023-07-14

**Authors:** Ovokeroye A. Abafe, Stuart Harrad, Mohamed Abou-Elwafa Abdallah

**Affiliations:** School of Geography, Earth and Environmental Sciences, University of Birmingham, Birmingham B15 2TT, United Kingdom

**Keywords:** microplastics, additive chemicals, polybrominated diphenyl ethers, hexabromocyclododecane, dermal bioaccessibility, cosmetics, particle size

## Abstract

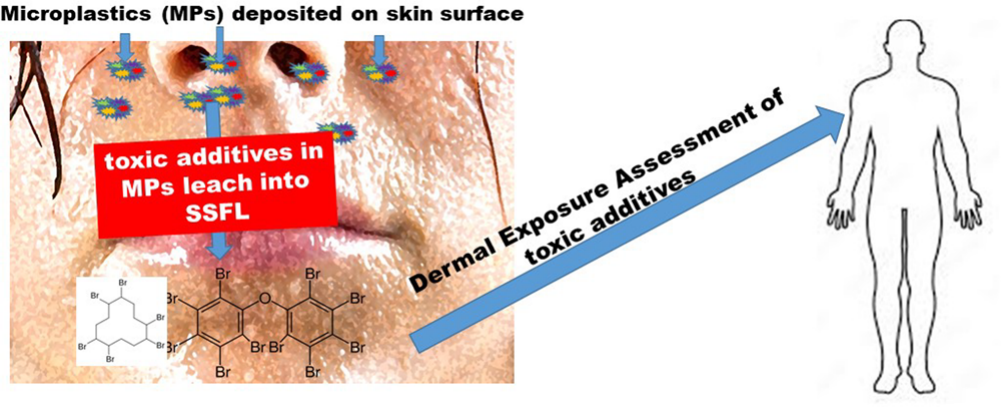

In this study, we optimized and applied an *in vitro* physiologically based extraction test to investigate the dermal bioaccessibility of polybrominated diphenyl ethers (PBDEs) and hexabromocyclododecane (HBCDD), incorporated as additives in different types of microplastics (MPs), and assess human dermal exposure to these chemicals. The dermal bioaccessibility of PBDEs in polyethylene (PE) MPs was significantly higher (*P* < 0.05) than in polypropylene (PP) MPs. Both log *K*_ow_ and water solubility influenced the dermal bioaccessibility of PBDEs. For HBCDDs in polystyrene MPs, the dermally bioaccessible fractions were 1.8, 2.0, and 1.6% of the applied dose for α-, β-, and γ-HBCDDs, respectively. MP particle size and the presence of cosmetic formulations (antiperspirant, foundation, moisturizer and sunscreen) influenced the bioaccessibility of PBDEs and HBCDDs in MP matrices at varying degrees of significance. Human exposure to ∑PBDEs and ∑HBCDDs via dermal contact with MPs ranged from 0.02 to 22.2 and 0.01 to 231 ng (kg bw)^−1^ d^–1^ and from 0.02 to 6.27 and 0.2 to 65 ng (kg bw)^−1^ d^–1^ for adults and toddlers, respectively. Dermal exposure to PBDEs and HBCDDs in MPs is substantial, highlighting for the first time the significance of the dermal pathway as a major route of human exposure to additive chemicals in microplastics.

## Abstract 2

There are no data on dermal exposure to microplastics or assessment of the risk arising from such exposure. This study reports the bioaccessibility of brominated flame retardants in various types of microplastics upon contact with human skin surface film liquid with implications for human dermal exposure.

## 1 Introduction

Microplastics (MPs) have been widely reported to be present in the marine and freshwater environment with concentrations up to 102000 particles m^–3^ in seawater and are also detected in sediment, fish, soil, dust, air, food, and drink.^1−4^ Such ubiquitous distribution of MPs in the environment and consumer products inevitably leads to human exposure to these particles, which has been confirmed by the recent detection of MPs in human blood,^5^ lungs,^6^ and stool.^7^ Meanwhile, animal studies have indicated MP exposure to elicit reproductive toxicity in oysters, reduced feeding in daphnia, and hepatotoxicity in zebrafish, as well as tissue accumulation and disturbance of lipid metabolism in mice.^6^ However, the toxicological impacts of human exposure to MPs are not well understood, which *inter alia* may be attributed to ethical constraints, strict biosecurity measures to handle human samples, and limited analysis techniques.

While particle exposure may lead to inflammatory lesions, bioaccumulation, and oxidative stress, there is also concern over the potential release of toxic chemical additives/adsorbed contaminants from MPs to human body fluids due to their small size and corresponding larger surface area.^1^ A wide range of chemical additives such as flame retardants, plasticizers, pigments, and fillers are often incorporated in plastics during manufacture to impart specific properties. Most of these additives, particularly in the flame retardants group, e.g. polybrominated diphenyl ethers (PBDEs) and hexabromocyclododecane (HBCDD), have been found to cause adverse health effects including endocrine disruption, reproductive toxicity, neurotoxicity, hepatotoxicity, and cancer.^8^ A recent study reported the leaching of toxic brominated flame retardants (BFRs) from acrylonitrile-butadiene-styrene copolymer (ABS) plastics to the *in vitro* simulated digestive fluids of birds, rendering them available for absorption, *i.e. bioaccessible*;^2^ however, there is no available information for humans. *Bioacessibility* is defined as the total amount of a chemical that is released from a solid matrix (e.g., dust, soil, MPs) into human or animal body fluids (e.g., saliva, gastric fluid, sweat) and thereby becoming available for absorption to the systemic circulation.^9^ In particular, there are no available data on the release and subsequent dermal uptake of toxic additive chemicals (e.g., flame retardants and plasticizers) to human skin surface film liquid (SSFL) upon contact of MPs with the skin, which is the largest body organ.^10^ The human SSFL consists of a mixture of sweat and sebum at various proportions.^11^ Sweat is an aqueous-based fluid secreted from sweat glands to regulate body temperature. It consists mainly of electrolytes, amino acids, organic acids, vitamins, and other nitrogenous substances. Sebum is a clear, oily liquid secreted from sebaceous glands to protect the skin from drying out. It mainly consists of triglycerides, squalene, wax esters, and fatty acids, with a small amount of cholesterol and cholesteryl esters.^12^

Dermal risk assessment has been traditionally based on the quantitative structure–activity relationship (QSAR)^13−15^ and pharmacokinetic (PK) modeling.^16^ These methods are limited by uncertainties related to (a) the fraction of chemicals available for absorption in PK modeling (i.e., bioaccessible fraction), (b) the partitioning between exposure doses in various solvent vehicles and the stratum corneum (the uppermost layer of the skin) especially for nonaqueous exposure vehicles, and (c) chemical diffusivity through the skin owing to differences in thickness of the stratum corneum among species.^17^*In vitro* physiologically based extraction tests (PBETs) have emerged as realistic alternatives to the traditional methods for assessing the bioaccessibility of hazardous chemicals from solid matrices and have been incorporated in regulatory risk assessment frameworks (BS EN 1811, 2011) and applied successfully to study the bioaccessibility of polychlorinated biphenyls (PCBs)^18^ and BFRs in house dust.^17^

The current understanding is that human exposure to MPs occurs through a combination of ingestion, inhalation, and dermal contact due to the presence of MPs in indoor dust, consumer products, water, foodstuffs, and air.^19^ While preliminary assessments of human exposure to MPs via ingestion and inhalation exist,^19^ there are no data on dermal exposure to MPs or assessment of the risk arising from such exposure. Recent studies by our research group highlighted the significance of dermal contact with synthetic furniture fibers as a major contributor to human body burdens of PBDEs and HBCDD^20^ and chlorinated organophosphate flame retardants.^21^ The adverse effects of flame retardants, combined with widespread dermal contact with MP particles (e.g., present in dust adhering to skin, atmospheric deposition of synthetic microfibers, and microbeads in personal care products), highlight the crucial need for further research into this area. Consequently, the current study assesses, for the first time, the dermal bioaccessibility of PBDEs and HBCDD incorporated in microplastics upon contact with synthetic human skin surface film liquid (i.e., sweat–sebum mixture). The factors influencing dermal bioaccessibility of these BFRs from MPs are evaluated, including: MP polymer type and particle size, BFR-specific physicochemical properties, and impact of topically applied cosmetics. Finally, the estimated bioaccessible fraction of the studied BFRs was applied to assess human dermal exposure to these toxic additive chemicals from various MP types, including polyethylene (PE), polypropylene (PP), and polystyrene (PS), using realistic but conservative exposure scenarios.

## 2 Materials and Methods

### 2.1 Chemicals and Reagents

All solvents used for sample preparation and gas and liquid chromatographic analysis were of HPLC grade (Fisher Scientific, Loughborough, United Kingdom). Individual standards of BDEs 28, 47, 99, 100, 153, 154, 183, 209, 77, 128, ^13^C_12_-BDE 100, and ^13^C_12_-BDE 209, as well as single standards of α-, β-, and γ-HBCDD, *d*_18_-γ-HBCDD, and ^13^C_12_-α-, β-, and γ-HBCDD were purchased from Wellington Laboratories Inc., Ontario, Canada. Two standard reference materials, European Reference Material for polyethylene (ERM-EC-590) and polypropylene (ERM-EC-591) certified for polybrominated diphenyl ether (PBDE) concentrations, were purchased from the European Joint Research Centre Institute for Reference Materials and Measurements (Brussels, Belgium). An in-house laboratory reference material of extruded polystyrene with known concentrations of α-, β-, and γ-hexabromocyclododecane (HBCDD) was obtained from the National Institute for Environmental Studies (NIES, Tsukuba City, Ibaraki, Japan). Cosmetics including sunscreen, antiperspirant, moisturizing cream, and foundation were of popular commercial brands purchased from a local UK supermarket.

### 2.2 Microplastic Samples

Microplastics from ERM-EC-590 and ERM-EC-591 with an original pellet particle size of approximately 4 mm were used in the study with MPs of particle size <0.45 mm produced from the original pellets using a Fritsch Pulverisette 0 cryo-vibratory micro mill (Idar-Oberstein, Germany). Frozen (−80 °C) plastic pellets of approximately 4 mm were placed in the stainless steel grinding mortar (50 mL volume) together with a 25 mm diameter stainless steel ball and submerged in liquid nitrogen (−196 °C) to aid the pulverisation process. The sample was ground at a vibrational frequency of 30 Hz for 5 min and repeated 3 times, resulting in plastic particles that passed through a 0.45 mm mesh aluminum sieve.

Extruded polystyrene samples were cut into small pieces, grated with a fine particle diameter stainless steel kitchen grater, and filtered through a 0.45 mm mesh stainless steel sieve. Two particle sizes of 3.5–4 mm (original pellet size provided by the manufacturer) and <0.45 mm were used for polyethylene and polypropylene MP experiments, while extruded polystyrene of particle size <0.45 mm was used for experiments related to HBCDDs.

### 2.3 Preparation of Synthetic Sweat and Sebum Mixture

The preparation of a physiologically simulated synthetic sweat and sebum mixture (SSSM) was conducted according to a US patent (US20080311613A1) using more than 25 organic and inorganic components (see Table S1 in the Supporting Information).^12^ The pH was adjusted to the physiological pH of the human skin surface film liquid (SSFL) (5.3 ± 0.1). Synthetic sweat and sebum mixtures were individually prepared and then mixed in different physiologically relevant ratios (Table S2) using drops of Tween-80 to mimic the naturally secreted SSFL and prevent phase separation.^22,23^

### 2.4 Physiologically Based Extraction Test (PBET) Protocol

The PBET protocol in this study was adopted from Pawar et al.^17^ and Ertl and Butte.^18^ Briefly, approximately 60 mg of microplastic sample (PE, PP, and PS) and approximately 6 mg of cosmetic (when tested) were weighed into a predried and sterilized glass test tube. Since no definitive data on the ratio of MPs to SSSM are available, we adopted a ratio of 1:100 w/v MP to SSSM ratio (i.e., 60 mg of each MP to 6 mL SSSM) to mimic “wet skin conditions” as previously reported.^17^ The mixture was gently agitated on a magnetic stirrer hot plate maintained at the physiological temperature of the human skin (32 ± 3 °C). Following 1 h of agitation, the mixture was phase-separated by centrifugation at 3500 rpm for 10 min. The supernatant (SSSM) and the solute (MPs) were analyzed separately. All experiments were carried out in triplicate. Various physiologically relevant SSFL compositions (i.e., different sweat:sebum ratios) were tested (Table S2).

### 2.5 Sample Extraction and Cleanup

Sample extraction and cleanup were conducted according to a previously reported method^24^ with slight modification. Briefly, each sample (PE and PP) was spiked with 100 ng of internal (surrogate) standard mixtures (BDE 128, ^13^C_12_-BDE 100 and -BDE 209), while PS samples were spiked with 60 ng of a ^13^C_12_-α-, β-, and γ-HBCDD internal standard mixture. This was followed by the addition of 3 mL of dichloromethane (DCM). The mixture was vortexed for 2 min followed by ultrasonication for 5 min and then centrifuged at 3500 rpm for 5 min. The organic phase was collected into a separate precleaned and sterilized glass test tube. The procedure was repeated twice. The collected extracts were evaporated to approximately 2 mL under a gentle stream of nitrogen set at 40 °C. 2 mL of hexane was added to precipitate any dissolved plastic and then reduced to approximately 1 mL and reconstituted in 2 mL of hexane to completely remove DCM followed by vortex mixing. Approximately 3 mL of concentrated sulfuric acid was added to samples and then vortexed for 1 min. The mixtures were left to stand for at least 5 h and then centrifuged at 3500 rpm for 5 min for phase separation. The organic layer was collected into a clean test tube. The sulfuric acid phase was further extracted twice with the addition of 2 mL of *n*-hexane, vortexed for 2 min, and centrifuged at 3500 rpm for 5 min. All the organic phases were combined and reduced to incipient dryness under a gentle stream of nitrogen at 40 °C. The extracts for PBDE analysis were reconstituted with 150 μL of isooctane containing 250 pg μL^–1^ BDE-77 as a recovery determination (syringe) standard (RDS), while the extracts from the PS (i.e., for HBCDD analysis) were reconstituted in 150 μL of methanol containing 250 pg μL^–1^ d_18_-γ-HBCDD. Full details of the extraction protocol for each type of sample are presented in the Supporting Information.

### 2.6 Instrumental Determination of PBDEs and HBCDDs

The operating conditions of the GC-MS method (for PBDEs analysis) and LC-MS/MS method (for HBCDDs analysis) used in the current study have been reported previously.^24,17^ Details of all instrumental parameters are presented in Table S3 in the Supporting Information.

### 2.7 Quality Assurance/Quality Control

A procedural blank containing SSSM without MPs was analyzed with every batch of 5 samples. None of the cosmetics were found to contain BFR concentrations above the LOQ of the target compounds; hence, results were not blank corrected. Method limits of detection (LODs) and limits of quantification (LOQs) were estimated based on S/N = 3:1 and 10:1, respectively. The LODs and LOQs ranged from 0.54 to 3.13 μg kg^–1^ and 1.87 to 9.50 μg kg^–1^ for PBDEs, while they ranged from 0.64 to 1.72 μg kg^–1^ and 1.94 to 5.22 μg kg^–1^ for HBCDDs, respectively.

The recovery of internal surrogate standards ranged from 115 to 117%, 61 to 97% and 40 to 127% for ERM-EC-590, ERM-EC-591, and ERM-EC-590 + cosmetics, respectively, while they ranged from 89 to 99% and 48 to 129% for PS and PS + cosmetics, respectively. To test the effectiveness of the method on the three MPs matrices following the bioaccessibility experiment, a mass balance was carried out as the sum of the concentrations of PBDEs and HBCDDs in the SSSM and in the residual MPs relative to the original concentrations determined in the MP matrices prior to bioaccessibility experiments. The mass balance recovery of PBDEs ranged from 80 to 152% and 56 to 103%, respectively, in PE and PP MPs at the physiologically relevant sweat:sebum (i.e., 1:1 sweat: sebum) mixture (Figure S1 in the Supporting Information). Whereas the mass balance recovery of HBCDDs ranged from 67 to 94% in polystyrene (Figure S2 in the Supporting Information), indicating the effectiveness of the analytical method for the determination of the target analytes in the respective matrices. Further quality control measures detailing the validation and reliability of the analytical method in this study are provided in Tables S4–S7 in the Supporting Information).

### 2.8 Bioaccessibility and Human Dermal Exposure to Additive BFRs in MPs

The bioaccessible fraction of each target chemical from each MP type is calculated from eq 1
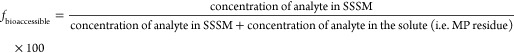
1where the concentration of analyte in SSSM is the amount of chemical released into the synthetic sweat:sebum mixture following the bioaccessibility experiment and the concentration of analytes in the solute is the amount of chemical remaining in the MP particles after the bioaccessibility experiment.

In assessing human dermal exposure to BFRs present as MP additives in the indoor environment, we employed 0.12 and 0.002 weight fractions of MPs in indoor dust (i.e., 0.12 g MPs/g dust for high-exposure and 0.02 g MPs/g dust for low-exposure scenario), as reported previously.^25^ Other factors used for the assessment of the dermal exposure dose (DED) of BFRs in MPs were obtained from the USEPA exposure factor handbook^26^ as presented in Table S8 in the Supporting Information.

The human dermal exposure dose (DED) to the target BFR was estimated using eq 2(26)
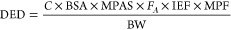
2where DED = daily exposure dose (ng kg^–1^ bw day^–1^), *C* = concentration of BFRs in MPs (ng/g), BSA = body surface area exposed (cm^2^), MPAS = MPs adhered to skin (mg/cm^2^), *F*_A_ = bioaccessible fraction (unitless), IEF = indoor exposure fraction (hours spent over a day in an indoor environment) (unitless), MPF = fraction of MPs in indoor dust (unitless), and BW = body weight (kg).

### 2.9 Statistical Analysis

The distribution of the data in this study was tested with the Shapiro–Wilk test and was found to be normally distributed. Descriptive statistics (e.g., mean, standard deviation etc.) were generated using Microsoft Office Excel 2016. Parametric tests (e.g., analysis of variance, Pearson’s correlation, etc.) were performed with XLSTAT Version 2021.3.1. *p < 0.05* was deemed significant.

## 3 Results and Discussion

### 3.1 Dermal Bioaccessibility of Polybrominated Diphenyl Ethers in Polyethylene and Polypropylene Microplastics

The skin, being the largest organ of the human body, is well-known to protect against pollutants, bacteria, ultraviolet radiation, etc.; however, it is consistently exposed to a wide range of xenobiotics both intentionally through the application of cosmetics or drugs and unintentionally through exposure to indoor and outdoor particle-bound environmental pollutants.^27^ While the penetration of a xenobiotic compound through the human skin is established to follow a passive diffusion process governed mainly by compound-specific physicochemical properties,^28^ for chemicals bound to solid matrices (e.g., particulate matter, MPs), the chemical’s release from the matrix into the human body fluid (i.e., SSFL) can be more important.^18,29^ In other words, a chemical within a solid matrix (e.g., MPs) must become *bioaccessible* first, in order to be available for absorption depending on its ability to penetrate the skin barrier.^9^

**Figure 1:**
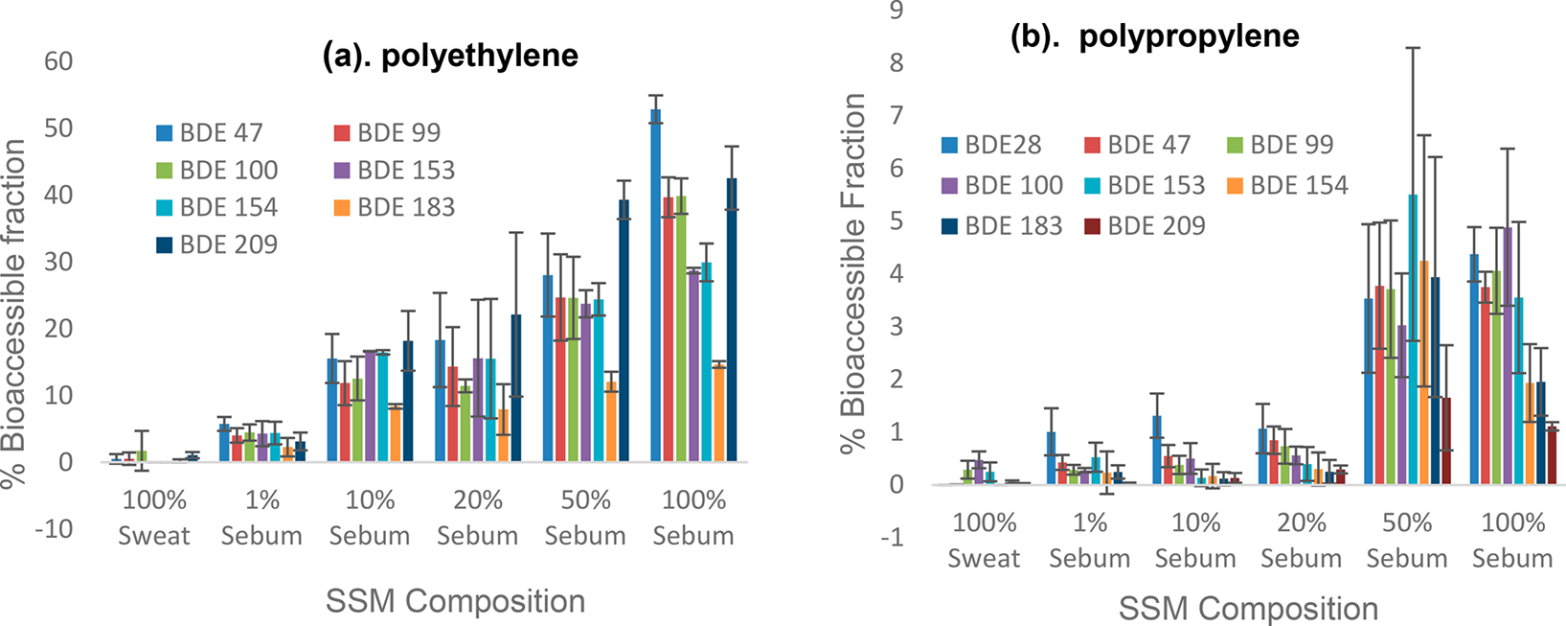
Impact of sweat:sebum composition on the dermal bioaccessibility of PBDEs in (a) PE MP pellets and (b) PP MP pellets.

The results of dermal bioaccessibility of PBDEs in polyethylene and polypropylene MPs are presented in Figure 1a,b. The bioaccessibility of PBDEs in PE and PP MPs increased with an increasing sebum ratio for all PBDE congeners. At the most physiologically relevant sweat: sebum composition (1:1), *f*_bioaccessible_ ranged from 1.7% for BDE 209 to 5.5% for BDE 153 and ∼12% for BDE 183 to ∼39% for BDE 209 in PP and PE microplastic pellets, respectively. These results prove, for the first time, the release of PBDEs from MPs into the SSFL, which is the first step in dermal uptake of these contaminants. BDE 209 (∼39%) was the most bioaccessible PBDE in PE MP pellets, with *f*_bioaccessible_ values of BDE-47, -99, -100, -153, and -154 ranging between 24 and 28%. The high bioaccessibility of BDE-209 may have been due to the higher concentration of this congener compared to the other BDE congeners in the PE and PP polymer matrices, which was at least 1 order of magnitude higher than the concentrations of most of the penta- and hexaBDE congeners, i.e. dose dependent. Also, BDE-209 being the most lipophilic of the PBDE congeners could have accumulated in the fat-rich (50%) synthetic skin surface film liquid. This is supported by the strong linear relationship between the bioaccessibility of PBDEs and fat contents in different food types reported by Yu et al.,^30^ as well as the observations of Hornero-Mendez and Minguez-Mosquera,^31^ in which the addition of olive oil to carrot prior to a bioaccessibility experiment significantly increased the % bioaccessible fraction of carotenoid (log *K*_ow_ 17.62).

The physicochemical parameters of PBDEs and HBCDDs are presented in Tables S9 and S10. The *f*_bioaccessible_ values for individual PBDE congeners in PE MP pellets showed moderate correlation (*p* < 0.05) with log *K*_ow_ (*r* = 0.45), log *K*_oa_ (*r* = 0.36) and log *K*_oc_ (*r* = 0.15). There were no significant associations between the bioaccessibility of the studied PBDE congeners and their water solubility or vapor pressure. Similarly, no association (*p* > 0.05) was observed between *f*_bioaccessible_ of PBDEs in PP MPs and their physicochemical properties (log *K*_oa_, log *K*_ow_, log *K*_oc_, water solubility, and vapor pressure). This indicates that while some physicochemical properties of PBDEs influenced their leaching from PE MPs, such influences were less influential drivers of the bioaccessibility of PBDEs in PP MP pellets.

The bioaccessibility of PBDEs in PE and PP microplastics differed significantly (*p* = 0.0002), with PE presenting higher *f*_bioaccessible_ values of PBDEs compared to PP MPs. This observation could be related to the lower relative density (0.92 g/cm^3^) of PP MPs, compared to PE MPs (0.97 g/cm^3^), which limits their sinking/rising behavior in liquid media, resulting in less interaction between the PP MPs and the SSFL. The decreased diffusivity of penetrants in polypropylene plastics has been previously noted.^32^ The diffusion coefficients of several hydrophobic organic compounds have been shown to be consistently lower in PP than in PEs,^33^ a phenomenon ascribed to the higher degree of branched chain carbons in PP. This is further exacerbated by the rigidity and the strong oil resistance properties of polypropylene plastics,^34^ which could repel the lipid components of human sweat:sebum mixtures.

Interestingly, the *f*_bioaccessible_ values of PBDEs in MPs in the present study were generally lower than the *f*_bioaccessible_ values reported for PBDEs in dust using an *in vitro* human gastrointestinal (GIT) PBET,^35^ with the exception of BDE 209, which was slightly more bioaccessible in our study. Overall, while the *f*_bioaccessible_ values of tri- to octaBDEs were generally higher in previous GIT PBET studies in indoor dust, the bioaccessibility of BDE 209 in MPs via the dermal pathway in the present study exceeded those estimated via the oral route using a colon-extended GIT model,^36^ dialysis membrane with the Tenax method^37^ and a fasting GIT model,^38^ highlighting the significance of dermal uptake of this lipophilic group of chemicals.^36−38^

#### 3.1.1 Impact of Particle Size on the Bioaccessibility of PBDEs in PE and PP Microplastics

**Figure 2:**
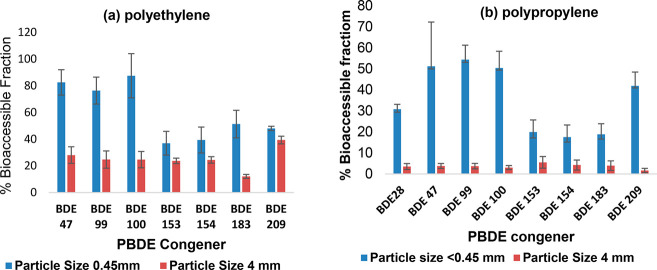
Impact of the particle size on the bioaccessibility of PBDEs in (a) PE and (b) PP MPs.

To determine the impact of particle size on the bioaccessibility of PBDEs in MPs, the original PE and PP MP pellets (3.5–4 mm) were crushed and passed through a 0.45 mm sieve. The bioaccessibility of tri- to octaBDE in the 0.45 mm MP particles thus generated was approximately double that in the original pellets. The *f*_bioaccessible_ values of PBDEs ranged from 12 to 39% and 37 to 82%, respectively, for the <4 and <0.45 mm polyethylene MP particles (Figure 2a). By comparison they ranged from 1.6 to 5.5% and 17 to 54% for the <4 and <0.45 mm polypropylene MP particles (Figure 2b), respectively. The *f*_bioaccessible_ values of PBDEs in the <4 and 0.45 mm fractions differed significantly (*p* < 0.05) in both the PE and PP MP particles. These differences could be due to the greater exterior surface area of the smaller particles coming into contact with the sweat:sebum mixture, leading to increased leaching of PBDEs into the skin surface film liquid. Our results highlight the impact of MP particle size and polymer type on the leaching behavior of PBDEs into human skin surface film liquid. This is consistent with the findings of Guo et al.,^2^ in which the leaching of PBDEs from plastics into the digestive fluids of birds increased with decreased particle size.

#### 3.1.2 Impact of Cosmetics on the Dermal Bioaccessibility of PBDEs from MPs

**Figure 3:**
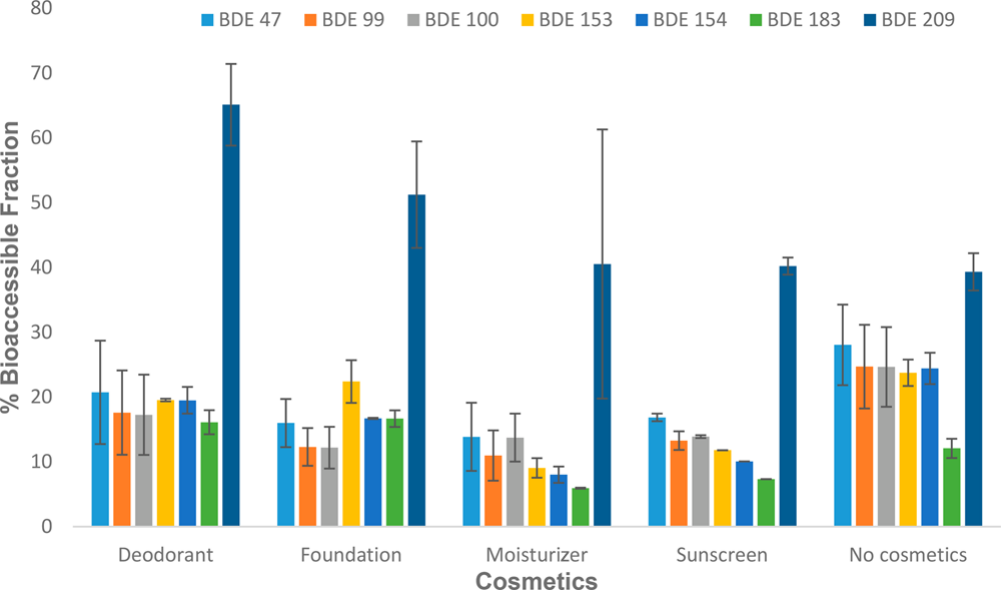
Impact of cosmetics on the bioaccessibility of PBDEs in MPs.

Cosmetics and their ingredients potentially remain on the skin for a long period of time and could alter the composition and physicochemical properties of the skin surface film liquid, which could potentially influence the bioaccessibility of chemicals from solid matrices in contact with skin.^17^ To investigate the influence of commonly applied cosmetics, we determined the *f*_bioaccessible_ values of PBDEs in PE and PP MPs into a 1:1 sweat:sebum mixture in the presence of antiperspirant, moisturizer, foundation, and sunscreen. The *f*_bioaccessible_ values of PBDEs in the presence of each of these cosmetics were compared with the *f*_bioaccessible_ value of the control group (i.e., same conditions but without any cosmetics). As shown in Figure 3, the bioaccessibility of BDE-209 increased with the introduction of antiperspirant and foundation but not with the moisturizer and sunscreen, which did not impact BDE-209 bioaccessibility. Though the cause of this observation is not fully understood, one plausible explanation could be that the ingredients in the foundations and antiperspirants impacted the surface tension of the sweat:sebum mixture, hence enhancing the solubility of the highly lipophilic BDE-209 in the 1:1 sweat:sebum mixture. This is supported by the strong positive correlation of the *f*_bioaccessible_ values of PBDEs following the application of antiperspirant and foundation with log *K*_ow_ (*r* = 0.86; *r* = 0.91) and log *K*_oc_ (*r* = 0.68; *r* = 0.77). This is in line with previous studies in which the active ingredients in sunscreen formulations were reported to enhance the dermal penetration of moderately lipophilic compounds, e.g. 2,4-dichlorophenoxyacetic acid.^39−41^

Conversely, the application of deodorant, foundation, moisturizer, and sunscreen inhibited the release of tri- to octaBDE from MPs into the sweat:sebum (1:1) mixture (Figure 3). Previous studies have shown that the presence of certain cosmetics decreased the bioaccessibility of polychlorinated biphenyls.^18^ However, the causes of these observations require further investigation.

### 3.2 Bioaccessibility of HBCDDs from Polystyrene MPs

**Figure 4:**
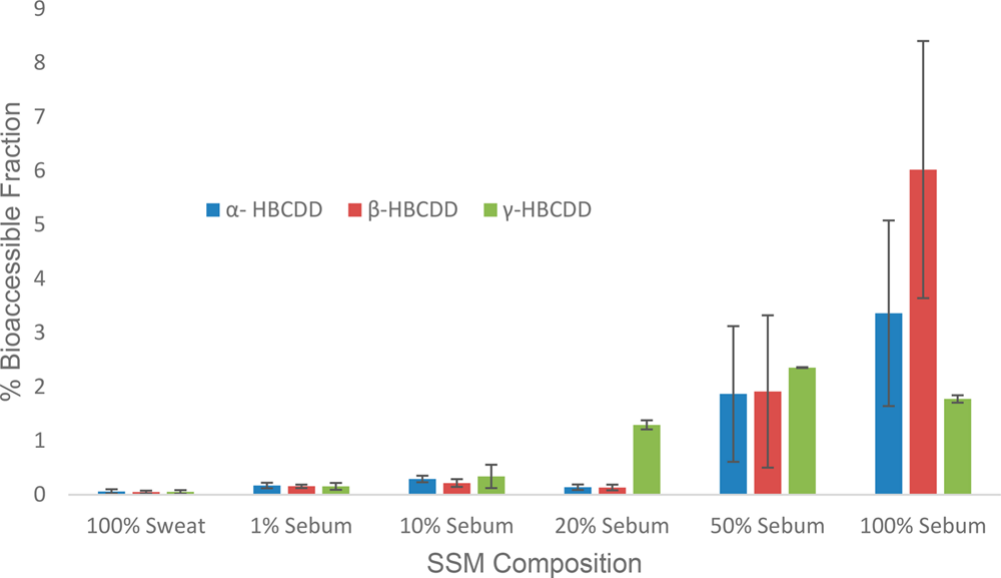
Influence of SSM composition on the bioaccessibility of HBCDD in polystyrene MPs.

The *f*_bioaccessible_ values of HBCDDs increased with an increase in sebum concentration. At lower sebum concentrations (i.e., at 100% sweat, 1% sebum, and 10% sebum), the *f*_bioaccessible_ value of HBCDDs in PS MPs was highest for the α-isomer followed by the β- and γ-isomers. However, the reverse was the case upon increasing the sebum composition from 20 to 50%, in which the γ-isomer was the most bioaccessible. At 100% sebum, the bioaccessibility of the β-isomer was highest, reaching 6% of the applied dose. Under the most physiologically relevant SSFL composition (1:1 sweat:sebum), the *f*_bioaccessible_ values were 1.6, 1.8, and 2.0%, respectively, for the α-, β-, and γ-HBCDD isomers (Figure 4). The bioaccessibility of HBCDDs showed a strong positive correlation with log *K*_ow_ (*r* = 0.999) and a fairly strong negative correlation with water solubility (*r* = −0.766), suggesting the influence of sebum on the release of HBCDDs from polystyrene MPs upon contact with the skin surface. In house dust, higher bioaccessibility of HBCDDs into a 1:1 sweat: sebum mixture (ranging from 41 to 50%) was reported previously.^17^ These values are an order of magnitude higher than the *f*_bioaccessible_ values for PS MPs in the current study. One plausible explanation for this observation would be the differences in matrices and less facile leaching of HBCDDs incorporated into PS microplastics than for HBCDDs adsorbed onto the surface of dust particles.

**Figure 5:**
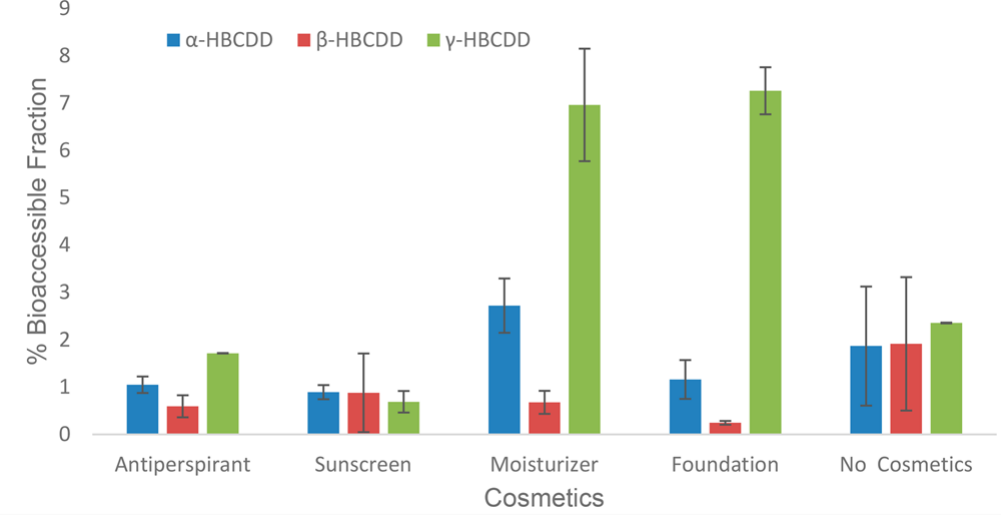
Influence of cosmetics on the bioaccessibility of HBCDDs in polystyrene MPs.

The impact of cosmetics on the bioaccessibility of HBCDDs from PS MPs varied for the different isomers. The application of moisturizer increased the *f*_bioaccessible_ values of α- and γ-HBCDD from 1.86 and 2.35% to 2.72 and 6.96%, respectively (Figure 5). Similarly, foundation cream increased the bioaccessibility of γ-HBCDD from 2.35 to 7.23% but decreased the *f*_bioaccessible_ values of α- and β-HBCDD from 1.86 and 1.91% to 1.16 and 0.25%, respectively. Both the sunscreen and antiperspirant decreased the *f*_bioaccessible_ values of all three HBCDD isomers. Although the application of cosmetics influenced the bioaccessibility of HBCDDs differently, only sunscreen significantly (*p* = 0.002) decreased the *f*_bioaccessible_ values of all 3 HBCDDs studied. Antiperspirant (*p* = 0.06), foundation (*p* = 0.72), and moisturizer (*p* = 0.49) did not significantly influence the bioaccessibility of HBCDDs. Similar decreases in the *f*_bioaccessible_ values of HBCDDs in house dust has been previously noted^17^—a phenomenon ascribed to the retention of lipophilic chemicals by lipids present in the cosmetics.

The *f*_bioaccessible_ values of HBCDDs following the application of cosmetics e.g. antiperspirant (*r* = 0.862), foundation (*r* = 0.905), and moisturizer (*r* = 0.972) showed strong correlation with isomer-specific log *K*_ow_ (Table S10).

### 3.3 Dermal Exposure to BFRs via Contact with MPs in Indoor Dust

The daily dermal exposure to PBDEs and HBCDDs (DED in units of ng (kg bw)^−1^ d^–1^) via contact with microplastics in indoor dust for adults and toddlers is presented in Table 1. PentaBDE congeners (BDE-47 and -99) and BDE-209 were the main contributors to the total exposure to PBDEs arising from dermal contact with PE and PP MPs in indoor dust. The daily exposure dose for adults ranged from 0.05 to 22.34 ng (kg bw)^−1^ d^–1^ and 0.001 to 0.37 ng (kg bw)^−1^ d^–1^ for the high and low MP exposure scenarios, respectively (i.e., 0.12 g MPs/g dust for high-exposure and 0.0 2g MPs/g dust for low-exposure scenario). While for toddlers, they ranged from 0.5 to 230 ng (kg bw)^−1^ d^–1^ and 0.01 to 3.85 ng (kg bw)^−1^ d^–1^, respectively, for high and low MP exposure scenarios. While the DED of PBDEs associated with polyethylene MPs exceeded their corresponding values in polypropylene MPs for the penta- and octaBDE congeners, polypropylene MPs delivered a higher exposure dose for BDE-209 in the indoor environment, albeit not statistically significant.

**Table 1:** Human Exposure to PBDEs (ng kg^–1^ bw d^–1^) via Dermal Contact with MPs at Home

	type of polymer											
	polyethylene				polypropylene				polystyrene			
	high intake (i.e., high MP intake)		low intake (i.e., low MPF)		high intake (i.e., high MP intake)		low intake (i.e., low MPF)		high Intake (i.e., high MP intake)		low intake (i.e., low MPF)	
BFR	adult	toddler	adult	toddler	adult	toddler	adult	toddler	adult	toddler	adult	toddler
BDE-28	0.2	2	0.004	0.01	0.1	0.5	0.001	0.01				
BDE-47	13	131	0.2	2	9	89	0.14	1.5				
BDE-99	14	143	0.2	2	12	123	0.20	2				
BDE-100	3	31	0.1	0.5	2.3	23	0.04	0.4				
BDE-153	1	13	0.02	0.2	0.6	6	0.01	0.1				
BDE-154	0.7	7	0.01	0.1	0.3	3	0.01	0.1				
BDE-183	2	20	1.9	0.3	1	12	0.02	0.2				
BDE-209	20	211	0.34	3.5	22	231	0.4	4				
α-HBCDD									2.8	52	0.1	0.5
β-HBCDD									1.1	12	0.02	0.2
γ-HBCDD									6.3	65	0.1	1.1

The DEDs of PBDEs in the current study were lower than the US EPA reference doses (RfD) of 2, 3, and 7 μg (kg bw)^−1^ d^–1^ for penta-, octa-, and deca-BDEs, respectively.^42^ While our exposure assessment for PBDEs in MPs was carried out with certified reference materials containing relatively high concentrations of PBDEs, higher concentrations of BDE-209 (260–2600 μg g^–1^) have been previously reported in indoor dust from UK and US homes that were identified to contain Br-rich polymer fragments.^43^ Another study also reported BFR concentrations ranging from 47000 to 69000 μg g^–1^ for BDE 209 in dust containing fibers and particles abraded from BFR-treated polymeric materials.^44^

For HBCDDs, DED values ranged from 1.13 to 6.27 ng (kg bw)^−1^ d^–1^ and 0.02 to 0.10 ng (kg bw)^−1^ d^–1^ for dermally exposed adults, while for toddlers, they ranged from 11.80 to 65.10 ng (kg bw)^−1^ d^–1^ and 0.20 to 1.08 ng (kg bw)^−1^ d^–1^ for the high- and low-exposure scenarios, respectively.

The results of the present study indicate that exposure to HBCDD via dermal uptake from MPs exceeds the lifetime average daily dose via dermal exposure through direct skin contact with flame-retarded curtains containing HBCDD concentrations an order of magnitude higher than those in the MPs in this study.^45^ Similarly, results from previous studies on HBCDD exposure via frequent hand-to-mouth contact were significantly lower than their DEDs arising from MP exposure.^46^ The present study highlights the significance of microplastics as a substantial source of human exposure to HBCDDs via the dermal pathway.

### 3.4 Study Limitations

This is the first study to experimentally assess human exposure to BFRs as chemical additives in MPs via any human exposure pathway; hence, it is not possible to compare the magnitude of dermal exposure from this pathway with other exposure pathways (e.g., inhalation and ingestion of MPs with BFR additives). While the DED values reported for these chemicals in this study highlight the significance of the dermal exposure pathway, the dermal exposure assessment in our study was conservative. We assumed that humans are only exposed to MPs via indoor dust containing as little as 0.2% (low-contact scenario) and 12% (high-contact scenario) of MPs^47^ with a conservative fraction of 1% of these values adhered to a fixed body surface area and using a fixed bioaccessible fraction of additive BFRs obtained from a 1 h experimental contact time. Also, the concentrations of BFRs in the certified reference materials are low compared with their concentrations in secondary MPs arising from flame-retarded plastics. These conservative scenarios are very unlikely, as the human skin is exposed daily to different kinds of MPs from various sources, including fabrics,^48^ personal care products,^49^ furniture,^50^ outdoor dust, and atmospheric deposition.^19^

While dermal contact with MPs occurs via a variety of sources, it is important to state that the dermal exposure estimate in this study was based solely on the bioaccessible fraction of these chemicals, which ranged from 1.56 to 2% for HBCDDs and 17 to 82% for PBDEs. These amounts of chemicals may not cross the stratum corneum to the epidermis and dermis into systemic circulation to exert toxic effects (i.e., bioavailable). In reality, the amount of these chemicals that would be available for systemic circulation would likely be considerably lower owing to the hydrophilic nature of the epidermis and dermis as previously reported for the lipophilic chemical benzo[*a*]pyrene, which penetrated easily into the lipidic layers of the stratum corneum but for which diffusion through the hydrophilic epidermis and dermis was low.^51^ This is consistent with previous results from our research group that established an increasing dermal resistance to the penetration of the more lipophilic γ-HBCDD compared to α- and β- isomers^28^ because as log *K*_ow_ increases, diffusive transport through the aqueous barrier becomes more restricted, thereby decreasing absorption.

To the best of our knowledge, the current study provides the first insights into the dermal bioaccessibility of additive BFR chemicals from different types of MPs and assesses the subsequent human dermal exposure to these chemicals via skin contact with MPs in house dust. We established various factors influencing the bioaccessibility of BFRs—a major class of additive chemicals used in various plastic polymers. These include the physicochemical properties of the studied BFRs, the polymer type, and MP particle size, as well as the use/application of various cosmetic formulations. We found that humans can be substantially exposed to target BFRs via dermal contact with flame-retarded MPs. We recommend that future studies should focus on the dermal bioavailability of these chemicals (i.e., their transfer across the skin barrier into the systemic circulation) for a more accurate quantification of the risk arising from dermal exposure to MPs. Moreover, it is important to establish the magnitude of human exposure to these toxic chemicals in MPs via other pathways e.g. ingestion and inhalation, in order to accurately examine the cumulative exposure dose and risk arising from such exposure.

## Supporting Information Available

Detailed description of the composition of the skin surface film liquids, detailed description of the extraction method and the optimized instrumental method parameters for gas chromatography-mass spectrometry and liquid chromatography with tandem mass spectrometry analyses, and detailed validation data for the experimental protocol and quality control (PDF)
