# Supplementary material for: Novel Insights into the Dermal Bioaccessibility and Human Exposure to Brominated Flame Retardant Additives in Microplastics
Source: Environ Sci Technol. 2023 Jul 14;57(29):10554–62. doi: 10.1021/acs.est.3c01894 (PMC10373483; doi:10.1021/acs.est.3c01894)
Supplement: Supplementary file 1 — es3c01894_si_001.pdf [file es3c01894_si_001.pdf]

Supplementary Information

Novel insights into the dermal bioaccessibility and human exposure to brominated flame retardant additives in Microplastics

*Ovokeroye A.Abafe[1,2], Stuart Harrad[1], Mohamed Abou-Elwafa Abdallah[1]

[1]School of Geography, Earth and Environmental Sciences, University of Birmingham, Birmingham B15 2TT, United Kingdom

*Correspondence: o.abafe@bham.ac.uk

(9 pages, 9 Tables, 2 Figures and 3 Texts).

Table S1. Sweat and sebum ingredients

Sweat Ingredients		
Ingredient	**Sweat**	**Sebum**
**Electrolytes and Ionic Constituents**	**Qty**	
Sodium Sulfate	0.0584 g	
Copper Chloride anhydrous	805 uL	
Ammonium Hydroxide	186	
Iron sulfate Heptahydrate	0.0028 g	
Lead- Reference Solution 1000 ppm	1243 uL	
Manganese- Reference Solution 1000 ppm	691.4 uL	
Nickel- Reference Solution 1000 ppm	1232 uL	
Zinc - Reference Solution 1000 ppm	1176 uL	
Sodium Bicarbonate	0.2530 g	
Potassium chloride	0.4550 g	
Magnesium Chloride Hexahydrate	0.0170 g	
Sodium Phosphate Anhydrous Monobasic	0.0484 g	
Calcium Chloride Dihydrate	0.7677 g	
Sodium chloride	0.0587 g	
**Organic Acids and Carbohydrates**		
Butyric Acid	220 uL	
Glucose	0.0294 g	
Lactic Acid	2011uL	
Essential Amino Acid Mix	4 mL	
**Nitrogenous Substances**		
Ammonium Chloride	0.0101 g	
Urea	0.5990 g	
Creatinine	0.0098 uL	
Squalene		0.25755 g
Palmityl Palmitate (saturated)		0.4859 g
Triolein (Unsaturated)		0.26725 g
Cholesteryl Oleate		0.0486 g

The volume of Milli Q water of about 70-80% of a final desired (1 L) liquid volume of the artificial sweat composition is taken and then the primary electrolytes and ionic constituents are added to form a primary solution. Then organic acids, amino acids, nitrogenous substances are added. The pH of the solution was adjusted to normal skin pH of 5.32 and preserved at 4 ± 3 °C.

Table S2: Sebum: sweat mixture (SSSM). $V_{total} = 100$ mL

% of sebum	Ratio (Sebum:Sweat)	Sebum (mL)	Sweat (mL)
0	0:100	0	100
1	1:99	1	99
10	10:90	10	90
20	20:80	20	80
50	50:50	50	50
100	100:0	100	0

Dissolve the sebum constituents in a sterilized 1000 mL Erlen Mayers flask containing 500 mL of jojobar oil with a magnetic stirrer bar.

The corresponding volume of sweat was first aliquoted into a sterilized 100 mL Schott followed by a dropwise addition of 50 µL of a non-ionic surfactant- Tween 80 with a constant agitation. Finally, the corresponding aliquot of the sebum was added slowly with rigorous agitation until a homogenous oil in water emulsion was formed. Both 20:80 and 50:50 SSSM were placed in an ultrasonic bath at 40 °C for 15 minutes to achieve sufficient homogenous solution. Each solution was vigorously shaken before use in bioaccessibility experiments.

Text S1. Sample extraction and clean-up for residues (i.e. plastics).

Each sample was spiked with 100 ng of internal (surrogate) standard mixtures (BDE 128, $^{13}C_{12}$ –BDE 100 and 209) followed by the addition of 3mL dichloromethane (DCM). The mixture was vortexed for 2 min followed by ultrasonication for 5 min and then centrifuged at 3500rpm for 5 min. The organic phase was collected into a separate pre-cleaned and sterilized glass test-tube. The procedure was repeated twice. The collected extracts were evaporated to approximately 2 mL under a gentle stream of nitrogen set at 40 °C. Two mL of hexane was added to precipitate any dissolved plastic and then reduced to approximately 1 mL and reconstituted in 2mL hexane to completely remove DCM followed by vortex-mixing. Approximately 3 mL of concentrated sulphuric acid was added to samples and then vortexed for 1 min. The mixture were left to stand for at least 5hr and then centrifuged at 3500 rpm for 5 min for phase separation. The organic layer was collected into a clean test tube. The sulphuric acid phase was further extracted twice with the addition of 2 mL n-hexane, vortexed

for 2 minutes and centrifuged at 3500 rpm for 5 min. All the organic phase were combined and blown to incipient dryness under a gentle stream of nitrogen at 40 °C. The extracts were reconstituted with 150 μL of isooctane containing 250 pg μL^{-1} BDE-77 as recovery determination (syringe) standard (RDS), while the supernatant (i.e. bioaccessible fraction) from the PS were reconstituted in 150 μL of methanol containing 250 pg μL^{-1} d18- γ-HBCDD.

For the PS residue, the sample was spiked with 60 ng $^{13}C_{12}$ α-, β- and γ-HBCDD followed by the addition of 3 mL DCM. The mixture was vortex for 2 min and sonicated for 5 min. The mixture was evaporated to approximately 2 mL and washed with 2 mL n-hexane and evaporated under a gentle stream of nitrogen at 40 °C to approximately 1 mL. Dissolved PS was precipitated with 2 mL n-hexane followed by the addition of approximately 3 mL sulphuric acid and then vortexed for 2 min. The mixture was left to stand for at least 5hr followed by centrifugation at 3500 rpm for 5 min. The organic layer was collected into a separate test-tube and the sulphuric acid phase was re-extracted twice with 2 mL n-hexane in each extraction cycle. All the organic layers were combined and evaporated to incipient dryness under a gentle stream of nitrogen at 40 °C. The extracts were reconstituted with 150 μL of methanol containing 250 pg μL^{-1} d18- γ-HBCDD.

Text S2. Instrumental determination of PBDEs and HBCDDs

Quantitative analysis of PBDEs was performed in a single injection on a Thermo Fisher Trace1310 gas chromatograph coupled to a Thermo Fisher ISQ mass spectrometer. The mass spectrometer was operated in electron ionisation mode using selective ion monitoring. One micro-litre of the purified extract was injected for analysis using a programmable temperature vaporiser onto a Restek Rxi-5Sil MS column (15 m×0.25 mm×0.25μm film thick-ness). Helium was used as the carrier gas at a flow rate of 1.5 mL/min with methane as the reagent gas.

HBCDDs were measured using a Shimadzu LC–20AB Prominence binary pump liquid chromatograph equipped with a SIL-20A auto-sampler, a DGU-20A3 vacuum degasser coupled to an AB Sciex API 2000 triple quadrupole mass spectrometer. Chromatographic separation was achieved using Agilent Pursuit XRS3 C18 column (150 mm×2 mm ID, 3μm particle size) and a mobile phase of (A) Water (B) Methanol at a flow rate of 180 μL/min. Molecular ionisation was achieved using an electrospray ionisation source operated in negative ion mode. Tandem MS/MS detection operated in the multiple reaction monitoring mode was used for quantitative determination of HBCDD isomers based on m/z 640.6→79,

m/z 652.4→79 and m/z 657.7→79 for the native, $^{13}C_{12}$-labelled and d_{18}-labelled diastereomers, respectively. The optimised LC–MS/MS parameters are summarised in Table S3.

Table S3: Optimized MS/MS parameters* for the analysis of HBCDs

Parameter	Value (units)
Curtain gas	35 (a.u.)
Turbo gas temperature	500 (°C)
Ion spray voltage	- 4500 (V)
Declustering potential	-5 (V)
Focusing potential	-365 (V)
Collision gas	5 (a.u.)
Collision energy	40 (eV)
Cell entrance potential	-6 (V)
Collision cell exit potential	-10(V)

a.u. – arbitrary units

* These parameters were obtained using direct infusion experiments of the target compounds (α-, β- and γ- HBCDs, native and ^{13}C- labelled isomers, 2 ng µL^{-1} each in MeOH) into the MS/MS system via a built-in Harvard syringe pump at a flow rate of 10 µL min^{-1}.

Text S3. Quality Control and Method Validation

Triplicate analysis of ERM-EC 590 and 591 as well as in-house extruded polystyrene reference materials were used to validate the method in this study. The measured concentrations were compared with the certified/known concentrations as presented in supplementary material Table S4. Overall, the analytical method deployed in this study satisfactorily measured the concentrations of the PBDEs and HBCDDs in the three types of microplastic (Polyethylene, Polypropylene and Polystyrene) matrices. To test the effectiveness of the method on the three MPs matrices following the bioaccessibility experiment, a mass balance was carried out as the sum of the concentrations of PBDEs and HBCDDs in the SSSM and in the residual MPs relative to the original concentrations determined in the MP matrices prior to bioaccessibility experiments. Figure 1 and 2 shows the recovery of PBDEs and HBCDDs respectively in MPs. The recovery of PBDEs ranged from 80 to 152 % and 56 to 103 %, respectively in PE and PP MPs at the physiologically relevant sweat: sebum (i.e. 1:1 sweat: sebum) mixture. Whereas the mass balance recovery of HBCDDs ranged from 67 to 94 % in polystyrene, indicating the effectiveness of the analytical method for the determination of the target analytes in the respective matrices.

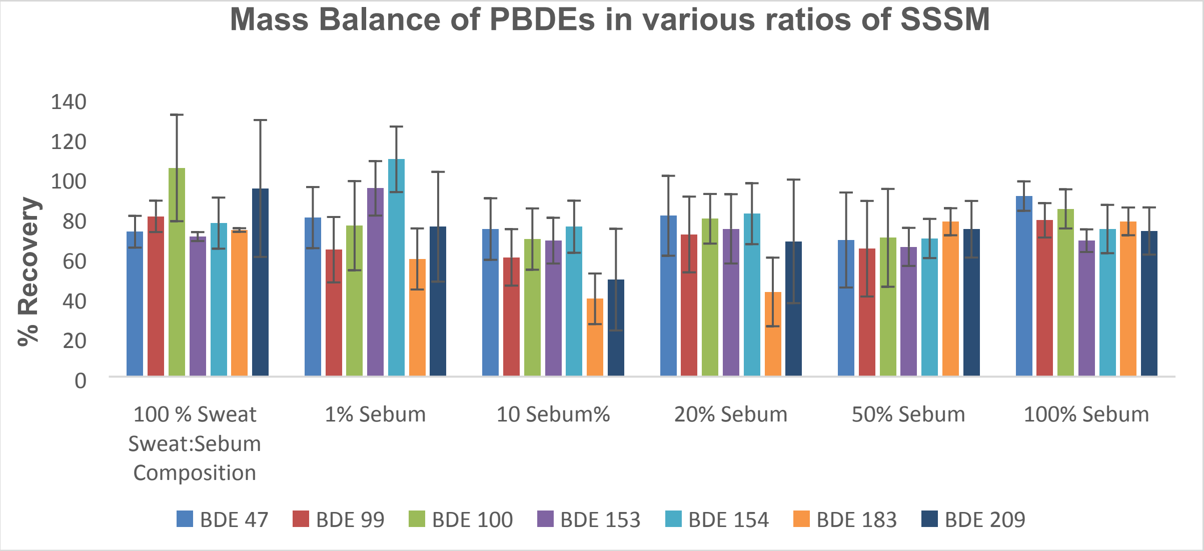

Figure S1. Mass Balance of PBDEs in Microplastics

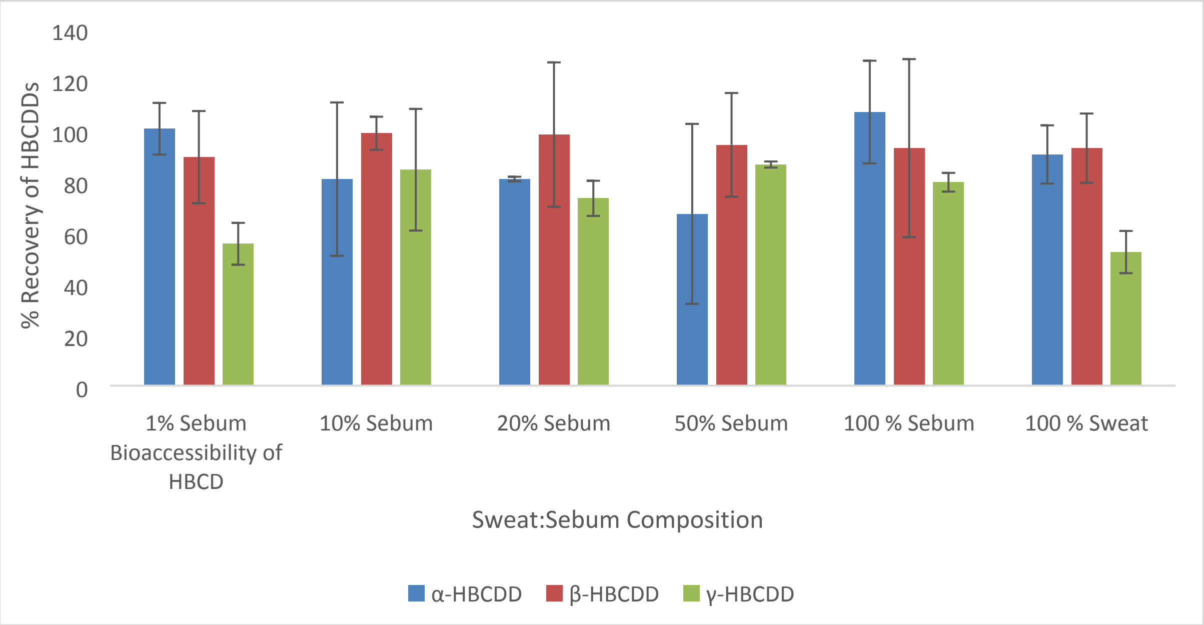

Figure S2. Mass balance of HBCDDs in Polystyrene

The recoveries of PBDEs and HBCDDs in the various cosmetic formulations are presented in supplementary Table S5 and S6, respectively. While the recoveries of internal standards in the matrices is presented in supplementary Table S7. Generally, the method displayed satisfactory performance in the analysis of the target compounds in the tested matrices.

Table S4. Validation of analytical protocol using standard reference materials

Analyte	ERM – EC590 (Polyethylene)		ERM – EC 591 (Polypropylene)		In-house certified Polystyrene	
	Certified Concentrations/ g kg^{-1}	Measured Concentration/ g kg^{-1}	Certified Concentrations/ g kg^{-1}	Measured Concentration/ g kg^{-1}	Certified Concentrations/ mg kg^{-1}	Measured Concentration/ mg kg^{-1}
BDE 28			0.00025	0.00017±0.00002		
BDE 47	0.23 ± 0.04	0.21 ± 0.006	0.245	0.217±0.027		
BDE 99	0.302 ± 0.030	0.25 ± 0.007	0.32	0.255±0.017		
BDE 100	0.063 ± 0.005	0.0472 ± 0.005	0.066	0.048±0.0001		
BDE 153	0.047 ± 0.006	0.028 ± 0.0010	0.044	0.065±0.0002		
BDE 154	0.0257 ± 0.0026	0.014 ± 0.0005	0.026	0.033±0.0001		
BDE 183	0.132 ± 0.010	0.68 ± 0.001	0.087	0.163±0.0007		
BDE 209	0.65 ± 0.10	0.93 ± 0.003	0.78	0.62±0.0002		
α - HBCDD					970	984.6 ± 136
β - HBCDD					620	498.7 ± 95
γ - HBCDD					3900	2632.69 ± 231

Table S5. Recovery of PBDE congeners in ERM 590 following the application of different cosmetics

	Deodorant	Foundation	Moisturiser	Sunscreen
BDE 47	90.18 ± 16.90	74.25 ± 3.74	74.34 ± 11.27	81.83 ± 0.77
BDE 99	80.22 ± 8.76	61.36 ± 5.10	63.57 ± 6.94	69.29 ± 2.19
BDE 100	84.73 ± 6.64	63.78 ± 3.77	69.69 ± 6.28	75.64 ± 1.26
BDE 153	88.34 ± 17.94	71.33 ± 7.46	71.65 ± 8.03	55.72 ± 18.5
BDE 154	86.43 ± 17.52	62.32 ± 7.51	67.32 ± 3.09	51.83 ± 19.6
BDE 183	69.90 ± 16.88	50.23 ± 2.40	52.92 ± 11.71	98.89 ± 1.94
BDE 209	79.68 ± 8.17	66.76 ± 9.40	70.16 ± 1.21	57 ± 5.29

Table S6. Recovery of HBCDDs in Polystyrene MP following the application of different cosmetics

	Antiperspirant	Foundation	Moisturiser	Sunscreen
α - HBCDD	73.76 ± 9.42	115.38 ± 19.79	119.09 ± 0.57	88.24 ± 0.15
β - HBCDD	65.77 ± 0.87	88.50 ± 18.83	73.82 ± 0.38	59.49 ± 23.87
γ - HBCDD	83.13 ± 36.10	86.52 ± 36.88	74.19 ± 17.27	80.77 ± 18.69

Table S7. Recovery of Internal Standards in the various matrices

BFR	α - HBCDD	β - HBCDD	γ - HBCDD	MBDE 100	BDE 128	$^{13}C_{12}$ – BDE 209
ERM-EC 590				115.49 ± 22.84	117 ± 23	116.43 ± 13.58
ERM-EC 591				61.08 ± 1.36	86.40 ± 10.97	97.48 ± 4.0
ERM 590 - ID				113 ± 29.07	79.22 ± 14.75	82.01 ± 24.80
ERM 590 - SC				68.59 ± 12.90	114.48 ± 4.21	94.14 ± 24.86
ERM 590 - SM				60.32 ± 7.6	126.96 ± 14.56	99.39 ± 27.45
ERM 590-TM				61.90 ± 7.63	40 ± 7.42	101.91 ± 15.52
PS	94.94 ± 11.78	99.21 ± 10.27	88.65 ± 13.70			
PS - ID	82.85 ± 0.17	82.13 ± 6.35	97.25 ± 2.31			
PS - SC	110 ± 25.95	84.20 ± 9.38	135 ±31.13			
PS - SM	129.14 ± 5.60	91.04 ± 9.66	48.06 ±10.73			
PS - TM	84 ± 8.30	56.98 ± 10.68	62.14 ± 2.71			

Table S8. Physicochemical properties of PBDEs

BDE	Water solubility mg/L (@25°C)	Log KOW	Log KOA (@25°C)	Log Koc	Vapour pressure (Pa) (@25°C)
BDE-28	0.07	5.94	9.5	3.91	0.000651
BDE-47	0.002	6.81	10.53	4.12	0.0000552
BDE-99	0.009	7.32	11.31	4.34	0.000000794
BDE-100	0.04	7.24	11.13	4.34	0.00000707

BDE-153	0.001	7.9	11.82	5.11	0.0000058
BDE-154	0.001	7.82	11.92	5.11	0.000000264
BDE-183	0.002	8.27	11.96	6.22	
BDE-209	0.001	10	13.21	6.8	9.28E-09

Table S9. Physicochemical properties of HBCDDs

	LogKow	Water solubility (mg/L)
α-HBCDD	5.07	48.8
β - HBCDD	5.12	14.7
γ - HBCDD	5.47	2.1
